# Contrast-Induced Encephalopathy following Cerebral Angiography in a Hemodialysis Patient

**DOI:** 10.1155/2020/3985231

**Published:** 2020-04-04

**Authors:** Marija Bender, Gojko Bogdan, Dorijan Radančević, Nataša Pejanović-Škobić

**Affiliations:** ^1^University Hospital Mostar, Department of Neurology, Mostar, Bosnia and Herzegovina; ^2^University Hospital Mostar, Department of Radiology, Mostar, Bosnia and Herzegovina

## Abstract

Contrast-induced encephalopathy (CIE) is a rare complication of contrast media use during angiographic procedures. With the growing use of endovascular interventions, this complication is likely to become more common. We present a case of a 46-year-old woman with hypertension, hypothyroidism, and chronic renal failure. She developed CIE following cerebral angiography for diagnosis of intracranial aneurysm. We had a high index of suspicion for CIE, excluded the most common differential such as stroke, and immediately started hemodialysis with a short course of corticosteroids. The disease runs a benign course, and neurological symptoms resolved completely after five days. We emphasize the need for increased awareness of CIE to make a valid diagnosis and to start supportive therapy as soon as possible.

## 1. Introduction

Contrast-induced encephalopathy (CIE) is a rare but well-known complication of contrast media use during angiographic procedures [1]. CIE was first reported in the 1960s [2]. The main features of this condition are transient cortical blindness, characterized by bilateral amblyopia or amaurosis, normal pupillary light reflexes, extraocular movements, and fundus [3]. Additional symptoms may include headache, memory loss, hemiparesis, aphasia, and reduction in higher mental function such as agraphia, loss of coordination, confusion, seizures, and coma [4]. Symptoms may begin during the procedure and up to 12 hours afterward, and they are usually self-limiting, resolving within 2–4 days of onset; rarely, complete recovery may take as long as few weeks [5, 6]. The disease typically runs a benign course; however, there have been reports of cases with persistent neurological deficit and even fatal outcome. Exact underlying mechanism of CIE remains unclear, and it likely relates to a transient breakdown of the blood-brain barrier with direct neurotoxicity of the contrast, which results in cerebral edema and alters neuronal excitability. This occurs most often in parieto-occipital lobes [7, 8]. Imaging in the form of CT or MRI scanning is essential to exclude this differential and confirm the diagnosis of CIE [5].

## 2. Case Report

**Figure 1:**
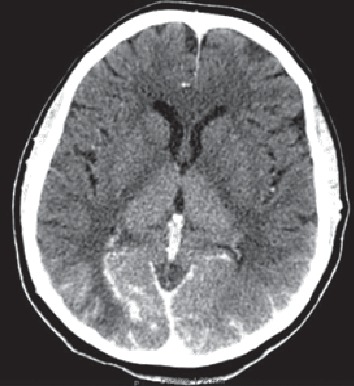
CT (axial) obtained one hour after DSA shows bilateral symmetrical contrast enhancement in parieto-occipital cortex and subarachnoid spaces, as well as in thalami.

**Figure 2:**
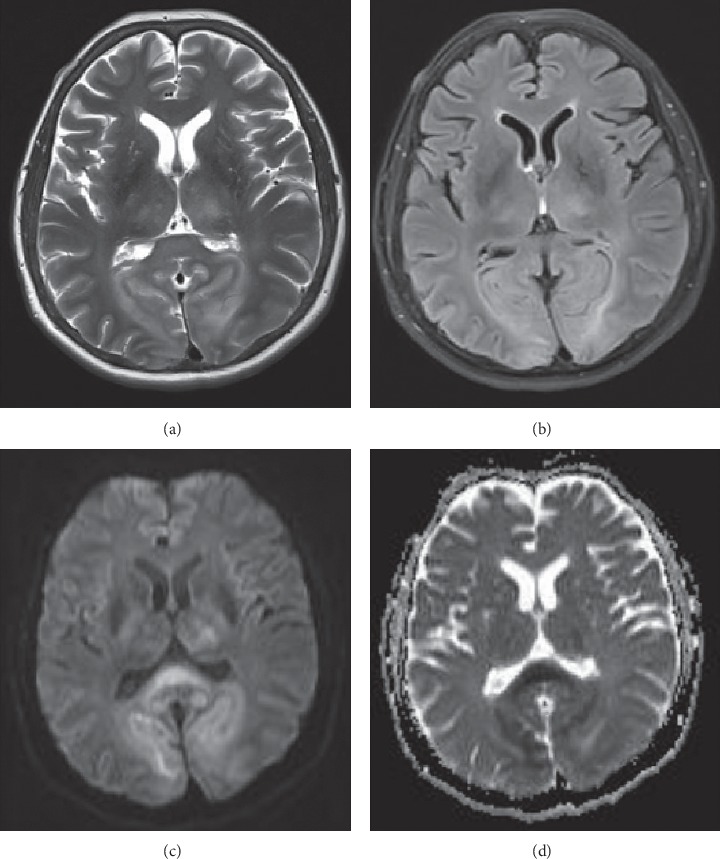
Brain MR (axial images) obtained 2 days after angiography. T2 and FLAIR images (a, b) show bilateral symmetrical hyperintensities in basal ganglia, in parieto-occipital cortex, and in splenium of corpus callosum. DWI and ADC images (c, d) demonstrate restricted diffusion in the same areas.

**Figure 3:**
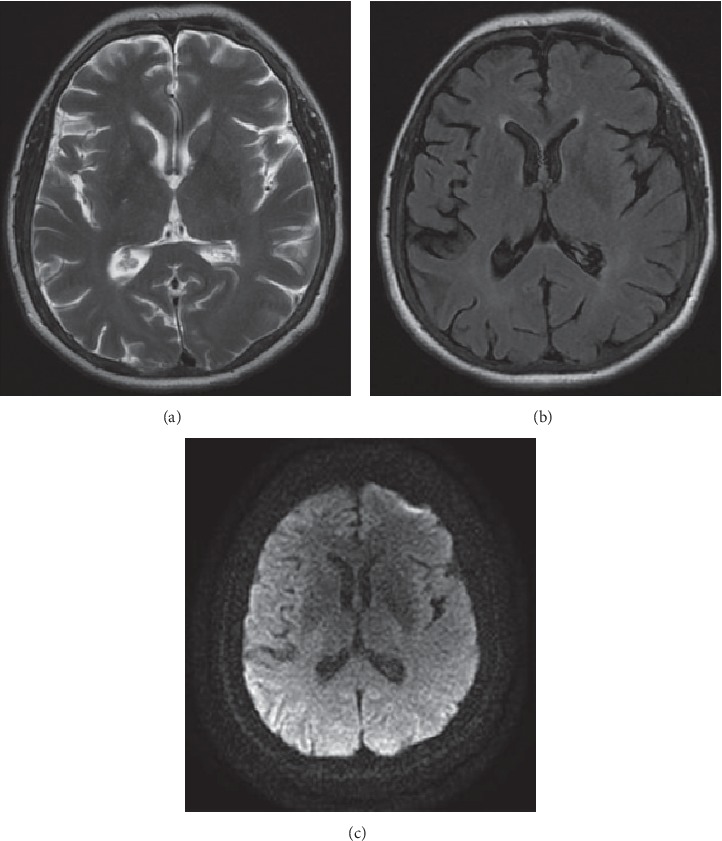
Follow-up brain MR-T2 (a), FLAIR (b), and DWI (c) axial images obtained in another facility 1 month after the initial MR shows complete resolution of the lesions.

A 46-year-old woman underwent cerebral angiography as a part of the diagnostic workup of an unruptured middle cerebral artery (MCA) aneurysm. An aneurysm of MCA, with a size of 15 mm × 12 mm, was detected on brain MRA during screening for headache. On admission, physical examination showed no signs of neurologic deficits. Her past medical history included hypertension, hypothyroidism, and polycystic kidney disease. Hemodialysis was initiated four years previously for chronic renal failure. She was a nonsmoker. Hemodialysis was performed on the day before the procedure. Under local anesthesia, diagnostic DSA showed a middle cerebral artery saccular aneurysm, with the size of 16 mm × 13 mm × 9 mm. The procedure lasted 20 minutes. A total of 80 ml of nonionic, hypoosmolar contrast medium “Iopamiro 370” (Iopamidol) was used, which is a standard in our hospital. This was the patient's first exposure to contrast medium. The contrast material was injected into the left and right carotid artery, as well as in the right vertebral artery, with no side effects noted. During injection of the contrast material into the left vertebral artery, the patient complained of blurring of vision which deteriorated to near-total blindness within minutes. The pupils were equal in size and responsive to light. The rest of the neurological examination was normal. Approximately 1 hour after the procedure, the patient developed an episode of a generalized tonic-clonic seizure. An emergency CT brain scan was requested and revealed bilateral symmetrical contrast enhancement in parieto-occipital cortex and subarachnoid spaces, as well as in thalami (Figure 1). Based on the CT brain scan, the presumed diagnosis was contrast-induced encephalopathy and hemodialysis was quickly performed. Later on, over the next 24 hours, she was confused and agitated, with severe headache, vomiting, and no signs of improvement. A brain MRI, performed on second postprocedure day, demonstrated T2 and FLAIR bilateral symmetrical hyperintensities in basal ganglia, in parieto-occipital cortex, and in splenium of corpus callosum, and the DWI and ADC images demonstrated restricted diffusion in the same areas (Figure 2). Daily hemodialysis was performed for the next 3 days, and she was treated with dexamethasone. Over the next few days, symptoms gradually started to improve progressing to complete recovery. On day 5, her neurological symptoms resolved completely. She was discharged home well. One month after discharge follow-up, brain MRI did not show any residual lesion, which correlates with the complete recovery (Figure 3).

## 3. Discussion

CIE is rare, but with the growing use of endovascular interventions as a diagnostic and a therapeutic means, this complication is likely to become more common. The initial diagnosis is challenging mostly due to its close resemblance to acute stroke. One should have a high index of suspicion in every patient with new-onset neurological deficit who underwent angiographic procedure, especially in patients with hypertension and renal failure [9]. Imaging in the form of CT or MRI scanning is important at the early stage of CIE. It is essential to exclude embolic or hemorrhagic complications, to avoid the administration of potentially harmful treatment such as thrombolysis and to initiate treatment at an early stage [5]. There is no consensus on the treatment of CIE, but the general advice, according to some authors, is the use of aggressive intravenous hydration and daily hemodialysis with a short course of corticosteroids [10, 11]. We emphasize the need for increased awareness of CIE to make a valid diagnosis and to start supportive therapy as soon as possible.
